# Design, synthesis and α-glucosidase inhibition study of novel embelin derivatives

**DOI:** 10.1080/14756366.2020.1715386

**Published:** 2020-01-22

**Authors:** Xiaole Chen, Min Gao, Rongchao Jian, Weiqian David Hong, Xiaowen Tang, Yuling Li, Denggao Zhao, Kun Zhang, Wenhua Chen, Xi Zheng, Zhaojun Sheng, Panpan Wu

**Affiliations:** a School of Biotechnology and Health Sciences, Wuyi University, Jiangmen, P.R. China; b International Healthcare Innovation Institute (Jiangmen), Jiangmen, P.R. China; c Department of Chemistry, University of Liverpool, Liverpool, UK; d School of Chemical Engineering and Light Industry, Guangdong University of Technology, Guangzhou, P.R. China

**Keywords:** Embelin, α-glucosidase inhibitor, anti-diabetes, hypoglycaemic agent, benzoquinone

## Abstract

Embelin is a naturally occurring *para*-benzoquinone isolated from *Embelia ribes* (Burm. f.) of the Myrsinaceae family. It was first discovered to have potent inhibitory activity (IC_50_ = 4.2 μM) against α-glucosidase in this study. Then, four series of novel embelin derivatives were designed, prepared and evaluated in α-glucosidase inhibition assays. The results show that most of the embelin derivatives synthesised are effective α-glucosidase inhibitors, with IC_50_ values at the micromolar level, especially **10d**, **12d**, and **15d**, the IC_50_ values of which are 1.8, 3.3, and 3.6 μM, respectively. Structure–activity relationship (SAR) studies suggest that hydroxyl groups in the 2/5-position of *para*-benzoquinone are very important, and long-chain substituents in the 3-position are highly preferred. Moreover, the inhibition mechanism and kinetics studies reveal that all of **10d**, **12d**, **15d**, and embelin are reversible and mixed-type inhibitors. Furthermore, docking experiments were carried out to study the interactions between **10d** and **15d** with α-glucosidase.

## Introduction

Diabetes mellitus (DM) is a group of chronic metabolic disorders characterised by hyperglycaemia resulting from defects in insulin secretion, insulin action, or both^1^^,^^2^. According to WHO data, DM was the 7th cause of death worldwide in 2016, when it killed 1.6 million people^3^. There are two major forms of the disease: Type 1 and Type 2 diabetes. The latter accounts for 90 ∼ 95% of all cases, formerly called non-insulin-dependent diabetes mellitus (NIDDM) or adult-onset diabetes, and usually occurs after age 40, becoming more common with increasing age^1^^,^^2^^,^^4^^,^^5^.

The management of Type 2 diabetes includes hyperglycaemia treatment, diabetic comorbidity prevention, and metabolism adjustment^6^^,^^7^. One therapeutic approach is to retard the absorption of glucose *via* inhibition of enzymes, such as α-glucosidase and α-amylase, in the digestive organs^8–11^. α-Glucosidase is an exo-type carbohydrase widely distributed in microorganisms, plants, and animal tissues. Inhibiting α-glucosidase slows the elevation of blood sugar after meals^8^^,^^12^. α-Glucosidase inhibitors (AGIs) are a unique class of oral hypoglycaemic agents approved for the prevention and management of Type 2 diabetes. Nowadays, there are four AGIs, including acarbose, miglitol, voglibose, and emiglitate, used in the clinical treatment of Type 2 diabetes^9^^,^^13^. However, current approaches including AGIs have some shortcomings such as safety concerns, limited efficacy, failure in metabolism adjustment, and the prevention of diabetic complications^6^^,^^9^^,^^14^. Thus, developing new therapeutic drugs to treat Type 2 diabetes is necessary, and has received wide attention.

Embelin is a naturally occurring *para*-benzoquinone isolated from *Embelia ribes* (Burm. f.) of the Myrsinaceae family, and contains two carbonyl groups, an active methylene group and two hydroxyl groups^15^^,^^16^. Embelin and its derivatives have been reported to possess anti-cancer^17^^,^^18^, antimicrobial^19^^,^^20^, antioxidant^21^, analgesic^22^, anti-inflammatory^22^, anxiolytic^23^, antifertility^24^ activities, etc. Thanks to these diverse biological activities, embelin is considered as the “second solid gold of India” next to curcumin^25^. In the last decade, several studies have reported antidiabetic activity of embelin^26–28^. In 2016, Sharanbasappa et al.^29^ reviewed the antidiabetic activity of embelin and its derivatives. It was concluded from this review and meta-analysis that the *E. ribes* extract, embelin and its derivatives have positive effects on blood glucose, HbAlc, insulin, and lipid profiles. In addition, heart rate, systolic blood pressure, lactate dehydrogenase, creatinine kinase and oxidative stress markers in diabetic rats return to normal after treatment with *E. ribes* extract and embelin. Moreover, Dang et al.^30^^,^^31^ reported that the methanolic extract of *E. ribes* and several compounds isolated from the leaves of *E. ribes* have significant α-glucosidase inhibitory activity. These results inspire us to investigate α-glucosidase inhibition of embelin, which is the main constituent of *E. ribes*. In this study, four series of novel embelin derivatives were designed and prepared in 5- to 6-step chemical reactions. Their α-glucosidase inhibitory activity was evaluated, and the mode of action and SAR analysis were described by means of kinetic and molecular modelling evaluations.

## Materials and methods

### Procedure for the synthesis of 1,2,4,5-tetramethoxybenzene (compound 3)

2,5-Dihydroxycyclohexa-2,5-diene-1,4-dione (5 g, 3.6 mmol) was dissolved in methanol (200 ml), and HCl solution (12 M, 6 ml) was added slowly. The mixture was stirred overnight. The reaction was monitored by TLC. After reaction completion, the mixture was filtered to obtain 2,5-dimethoxycyclohexa-2,5-diene-1,4-dione **1** as a yellow solid. Compound **1** was dissolved in H_2_O (50 ml), and Na_2_S_2_O_4_ (10 g, 57.4 mmol) was added. The mixture was heated to reflux for 8 ∼ 15 min. Then the mixture was cooled to crystallise, and filtered to furnish 2,5-dimethoxybenzene-1,4-diol **2** as a white crystalline solid. Compound **2** (1.7 g, 10 mmol) was dissolved in DMSO (10 ml), and KOH (1.4 g, 25 mmol) was added. The mixture was stirred for 15 min, and then MeI (1.88 ml, 25 mmol) was added. The mixture was stirred overnight. After reaction completion, the pH value was adjusted to 5 ∼ 6 by saturated NH_4_Cl solution. The mixture was washed with saturated NaCl solution and H_2_O and dried over MgSO_4_. The solution was condensed under reduced pressure to give crude product, which was purified by silica gel column chromatography (PE/EtOAc = 10/1), yielding 1,2,4,5-tetramethoxybenzene **3** (1.05 g, 53%).

**Scheme 1.**
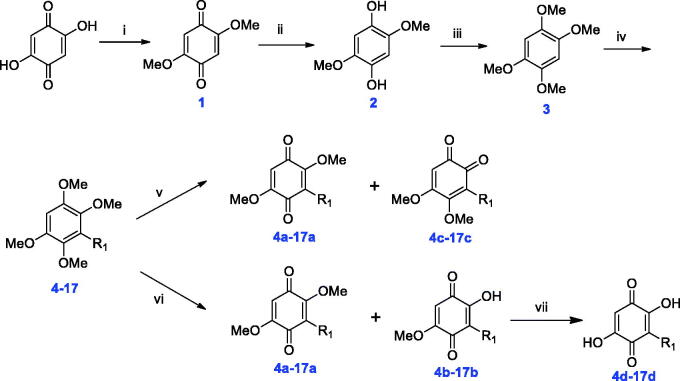
Synthesis of novel embelin derivatives.

Reagents and conditions: (i) conc. HCl, MeOH, rt., overnight; (ii) Na_2_S_2_O_4_, H_2_O, reflux, 8 ∼ 15 min; (iii) KOH, MeI, DMSO, rt., overnight; (iv) *n*-BuLi, HMPA, THF, −40 °C to rt., overnight; (v) CAN, MeCN/H_2_O, −5 °C, 10 min; (vi) CAN, MeCN/H_2_O, −5 °C to rt., 2 h; (vii) NaOH, EtOH, 80 °C, 2 ∼ 3 h.

### General procedure for the synthesis of 4–17

Hexamethylphosphoric triamide (HMPA, 353 μL, 2 mmol) was added to a solution of compound **3** (1 g, 5.1 mmol) in dry THF (60 ml) under N_2_ atmosphere. Next, *n*-BuLi (2.44 ml, 6.12 mmol) was added slowly to the mixture at −40 °C and stirred at this temperature for 10 min. Then the mixture was allowed to heat to −10 °C, and various halogenated hydrocarbons (5.5 mmol) were added dropwise. The mixture was stirred at room temperature overnight. THF in the reaction mixture was removed under reduced pressure, and the residue was redissolved in EtOAc. The organic solution was washed with 1 M HCl solution and saturated brine, then dried over MgSO_4_ and evaporated. The crude product was chromatographed (PE/EtOAc = 15/1 to 5/1) to supply pure compound **4–17** (20%∼60%).

### General procedure for the synthesis of 4a–17a, 4b–17b, and 4c–17c

A solution of ceric ammonium nitrate (CAN, 1.4 mmol) in MeCN/H_2_O (7/3, 5 ml) was added to a solution of compounds **4–17** (0.55 mmol) in MeCN (4 ml) in a salt-ice bath.

**Method A.** The suspension was stirred for 10 min at −5 °C. The organic solvent was removed and the residue was redissolved in EtOAc (5 ml). The organic layer was washed with saturated brine, dried over MgSO_4_ and condensed. The crude product was purified by flash chromatography (PE/EtOAc = 10/1), furnishing first pure **4a–17a** (32%∼40%). Then, the eluent was changed to PE/EtOAc = 5/1, giving pure **4c–17c** (23%∼30%).

**Method B.** The suspension was stirred for 2 h at room temperature. The organic solvent was removed and the residue was redissolved in EtOAc (5 ml). The organic layer was washed with saturated brine, dried over MgSO_4_ and condensed. The crude product was purified by flash chromatography (PE/EtOAc = 10/1), furnishing first pure **4a–17a** (35%∼43%). Then, the eluent was changed to PE/EtOAc = 3/1, giving pure **4b–17b** (18%∼27%).

### General procedure for the synthesis of 4d–17d

2 M NaOH solution (8 ml) was added to a solution of compounds **4a–17a** or **4b–17b** (0.35 mmol) in EtOH (2 ml). The mixture was heated to reflux for 2 ∼ 3 h. Next, the organic solvent was removed. The pH value of the aqueous solution was adjusted to 5 ∼ 6. Then, the solution was diluted with EtOAc (3 ml). The organic layer was washed with saturated brine, dried over MgSO_4_, and condensed. The crude product was then washed with a low-polar organic solvent to remove impurities, and filtered to obtain pure **4d–17d** (20%∼45%).

### α-Glucosidase inhibition

The α-glucosidase inhibitory activity was determined on a Thermo Scientific Multiskan GO Microplate Reader. All compounds were dissolved in DMSO. *p*-Nitrophenol glucoside (PNPG) as a substrate and α-glucosidase (from *Saccharomyces cerevisiae*) were used for the bioassay. First, 0.7 U/ml α-glucosidase solution and 1 mmol/L PNPG solution in 0.1 M PBS (pH 6.8) were prepared. Next, 35 µl of 0.1 M PBS (pH 6.8), 10 µl of 0.7 U/ml α-glucosidase solution (final concentration: 0.07 U/ml), and 5 µl of sample solution were added to the 96-well plate. The mixture was pre-incubated for 10 min at 37 °C. Then, 50 µl of 1 mM PNPG (final concentration: 0.5 mM) was rapidly added to initiate the reaction. Then the 96-well plate was quickly transferred into the microplate reader, incubated at 37 °C for 30 min (1 min shake + 9 min incubation, repeated 3 times). After that, 50 µl of 1 M of Na_2_CO_3_ solution was added to each well to stop the reaction. The 96-well plate was quickly transferred into the microplate reader and shaken for 30 s. In the blank reference group, 5 µl DMSO replaced the sample solution, other operations were the same. The OD value was measured at 405 nm. The inhibition rate was calculated by Equation (1).

(1) $$\text{Inhibition}\;\text{rate}\;\left(\%\right)\;=\;\left[\left(A_{0}-A_{1}\right)/A_{0}\right]\;\times \;100$$

where A_0_ is the OD of the control, and A_1_ is the OD of the sample.

The inhibition mechanism of **10d**, **12d**, and **15d** was determined by the following method: A series of diluted inhibitor solutions was prepared, at constant PNPG concentration (1 mM). The inhibition rates were measured by the above method with different concentrations of α-glucosidase (0.00, 0.35, 0.7, and 1.05 U/ml).

The inhibition type of the enzyme was assayed by Lineweaver–Burk plots. A series of diluted inhibitor solutions was prepared, at constant α-glucosidase concentration (0.7 U/ml). The inhibition rates were measured by the above method with different concentrations of PNPG (1, 0.5, 0.4, 0.3, 0.25, and 0.2 mM).

### Molecular docking

The molecular modelling and docking simulation were implemented by using Sybyl 2.0. The 3D structure of α-glucosidase (PDB code: 1UOK) was obtained from RCSB Protein Data Bank and the two most potent embelin derivatives (**10d** and **15d**) were built by molecular modelling package in Sybyl. All the water molecules were removed and hydrogen atoms were added to make protein and ligand protonation. Tripos force field was adopted to make the energy of protein and ligand to be minimised. Ligand–protein docking simulations were processed by the Surflex-Dock within Sybyl. The catalytic pocket of acarbose docking with α-glucosidase was defined as active site. Other settings were kept default.

## Results and discussion

### Compound design and synthesis

**Figure 1.**
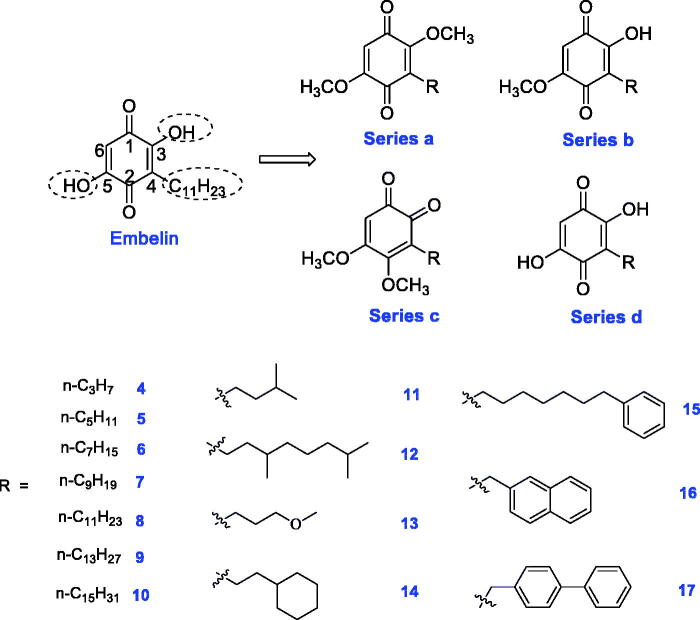
Design of novel embelin derivatives.

In our previous study, embelin displayed potent α-glucosidase inhibitory activity with an IC_50_ value of 4.20 μM. In order to investigate structure–activity relationships (SAR) and obtain more active AGIs, four series (**a**, **b**, **c**, and **d**) of novel embelin derivatives were designed (Figure 1). First, in order to study the function of the hydroxyl groups in the 2/5-position, they were monomethylated or dimethylated, giving series **a** and **b**. Second, the core structure was varied from *para*-benzoquinone to *ortho*-benzoquinone, giving series **c**. Third, because the long C_11_H_23_ tail in the 3-position results in the poor bioavailability of embelin, new groups were introduced at this position, giving series **d**. The structures of R groups are shown in Figure 1. In order to study the length of the substituents, C_3_- to C_15_-*n*-alkyl groups were introduced (Compounds **4 ∼ 10**). In addition to the straight-chain groups, branched alkyl groups or straight-chain groups containing a heteroatom were introduced. Moreover, aromatic rings or cycloalkanes were introduced in the terminal group.

The target compounds were prepared according to a published method^32^ with slight modification. 2,5-Dihydroxycyclohexa-2,5-diene-1,4-dione was used as starting material: first, the two hydroxyl groups were protected by methylation to yield compound **1**. Then the benzoquinone core structure was reduced by Na_2_S_2_O_4_, giving compound **2**. Further methylation produced 1,2,4,5-tetramethoxybenzene **3**. Next, *n*-BuLi was used to abstract the hydrogen atom from the benzene ring, and then various halogenated hydrocarbons were added to furnish **4 ∼ 17**. The yield of this step depended on the steric hindrance of the halogenated hydrocarbon. For example, we tried to introduce α-naphthyl into the core structure but failed due to its large steric hindrance. Although β-naphthyl and biphenyl were introduced successfully, the synthetic yields of **16** and **17** were low. The oxidation of **4 ∼ 17** by CAN produced different oxidised products depending on the reaction time. At the beginning of the oxidation reaction, a mixture of **4a∼17a** and **4b∼17b** was obtained. As the reaction proceeded, mixtures of **4a∼17a** and **4c∼17c** were obtained as the main products. Finally, the demethylation of **4a∼17a** and **4b∼17b** under basic conditions gave **4d∼17d**. The final products **4a∼17a**, **4b∼17b** and **4c∼17c** were purified by flash chromatography, and **4d∼17d** were purified by washing with petroleum ether or dichloromethane.

**Figure 2.**
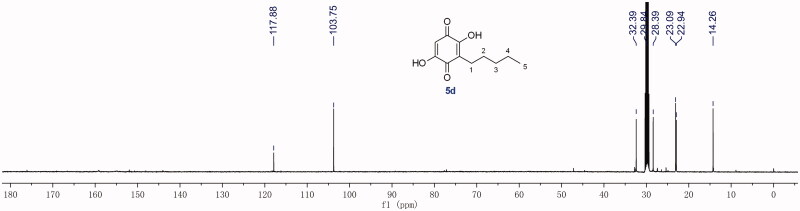
^13^C-NMR spectrum of compound **5d**.

**Figure 3.**
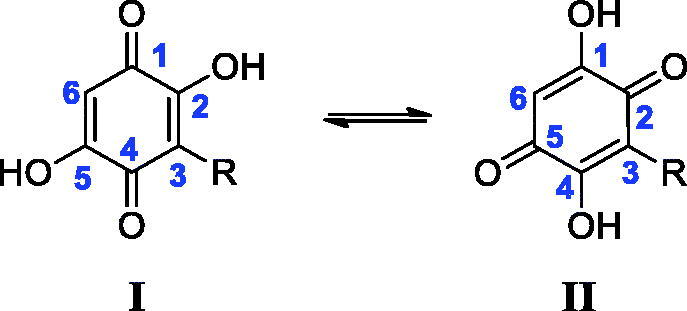
Tautomerism between two forms of *para*-benzoquinone.

All final compounds were characterised by ^1^H-NMR, ^13^C-NMR, and HRMS. It is noteworthy that there is an interesting phenomenon in the ^13^C-NMR spectra of compounds **4d∼17d**. Taking **5d** as an example (Figure 2), six different carbons exist in the *para*-benzoquinone ring. However, there are only two carbon signals at >90 ppm, which belong to the carbons in the 3/6-position. This may be due to keto-enol tautomerism of *para*-benzoquinone (Figure 3). This rapid transfer between two tautomers makes it difficult to record the corresponding carbon signals. Once one of the hydroxyl groups is methylated, the ^13^C-NMR signals of the core structure return to be normal.

### α-Glucosidase inhibitory activity and SAR analyses

The inhibitory activities of embelin and its derivatives on α-glucosidase from baker’s yeast were determined by the reported method^6^^,^^11^ with slight modification using acarbose and ursolic acid as the positive reference compounds. As depicted in Table 1, the inhibitory activities of all compounds were screened at the concentration of 250 μM. Most compounds showed an obvious inhibitory effect on α-glucosidase. Among the four series, compounds from series **d** showed much better inhibitory activity than those from the other three series, which indicates that the hydroxyl groups in the 2/5-positions are very important for the α-glucosidase inhibitory activity. Moreover, the modification of the core structure from *para*-benzoquinone to *ortho*-benzoquinone resulted in a significant reduction of activity.

**Table 1.** Inhibition rate of all compounds at 250 μM against α-glucosidase.

Comp.	Inhibition rate	Comp.	Inhibition rate	Comp.	Inhibition rate	Comp.	Inhibition rate
**4a**	9.0%	**4b**	10.4%	**4c**	14.9%	**4d**	18.1%
**5a**	11.5%	**5b**	14.9%	**5c**	16.4%	**5d**	19.0%
**6a**	1.5%	**6b**	5.4%	**6c**	16.1%	**6d**	80.8%
**7a**	13.5%	**7b**	20.9%	**7c**	33.2%	**7d**	99.4%
**8a**	12.5%	**8b**	12.0%	**8c**	0.6%	Embelin	100%
**9a**	nt	**9b**	nt	**9c**	nt	**9d**	100%
**10a**	nt	**10b**	nt	**10c**	nt	**10d**	100%
**11a**	1.5%	**11b**	2.6%	**11c**	3.3%	**11d**	37.6%
**12a**	nt	**12b**	nt	**12c**	nt	**12d**	100%
**13a**	0%	**13b**	0%	**13c**	0%	**13d**	0%
**14a**	70.1%	**14b**	71.1%	**14c**	74.7%	**14d**	85.2%
**15a**	nt	**15b**	nt	**15c**	nt	**15d**	100%
**16a**	6.0%	**16b**	4.3%	**16c**	37.2%	**16d**	81.4%
**17a**	5.1%	**17b**	14.3%	**17c**	59.7%	**17d**	99.5%

nt: not tested.

**Figure 4.**
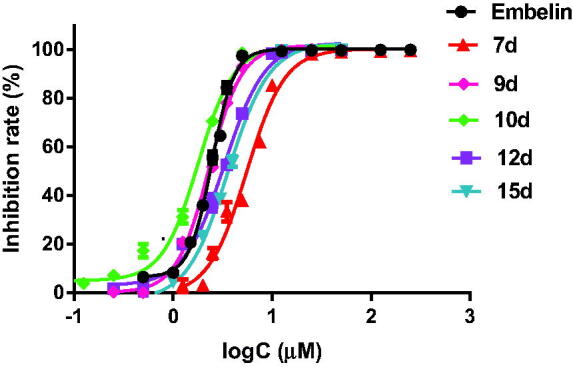
Inhibition curves of embelin and its derivatives.

Then, the inhibition rates of the compounds which inhibited more than 80% of α-glucosidase in the initial screening were further evaluated at more than eight concentrations. The IC_50_ values were calculated by GraphPad Prism 7.0 software. The results are summarised in Table 2 and the inhibition curves are shown in Figure 4. The length of the chain in the 3-position is a key factor affecting the activity. The compounds with longer chains display better activity. For example, compounds **4d** and **5d** have a very low inhibitory effect, while **6d∼10d** have potent activity, which suggests the length of the chain should be longer than *n*-C_7_H_15_. Moreover, when the length was similar, branched groups slightly enhance the activity. For example, the lengths of the chain in the 3-position of compounds **5d** and **11d**, **7d**, and **12d** were similar, but **11d** exhibited better inhibitory activity than **5d**, and **12d** showed slightly better activity than **7d**. However, the introduction of an oxygen atom in the chain results in the loss of activity (compounds **13a∼13d**). In addition, the introduction of large hydrophobic groups such as cyclohexyl, phenyl, β-naphthyl, and 4-biphenyl (compounds **14d**, **15d**, **16d**, and **17d**) in the terminal, seems to have positive influence on the activity. Meanwhile, the length of these large hydrophobic groups is closely related to the activity.

**Table 2.** IC_50_ values of selected compounds against α-glucosidase.


Compound	R	IC_50_ (μM)
6d	*n*-C_7_H_15_	126.8
7d	*n*-C_9_H_19_	5.7
embelin	*n*-C_11_H_23_	4.2
9d	*n*-C_13_H_27_	2.3
10d	*n*-C_15_H_31_	1.8
12d		3.3
14d		45.3
15d		3.6
16d		74.8
17d		11.2
Acarbose		584.0
Ursolic acid		4.3

### Inhibition mechanism

**Figure 5.**
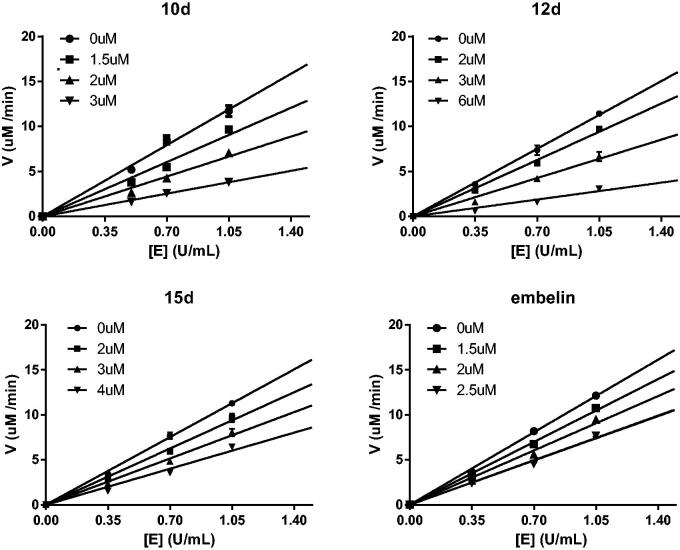
Determination of the mechanism of the inhibition of α-glucosidase by **10d**, **12d**, **15d**, and embelin.

Compounds **9d**, **10d**, **12d**, and **15d** displayed the most potent activity in the α-glucosidase inhibitory assays. Because the chemical structures of compounds **9d** and **10d** are very similar, compound **10d** was selected as a representative in the next mechanism studies. The inhibition mechanism of the selected compounds **10d**, **12d**, **15d**, and embelin on α-glucosidase was investigated using PNPG as the substrate. The relationship between enzyme activity and concentration in the presence of different concentrations of selected compounds was studied. As shown in Figure 5, the plots of enzyme activity *versus* enzyme concentration give a set of straight lines, which all pass through the origin. The increase in the inhibitor concentration resulted in the reduction of the slope of the line, which suggests that the inhibitors reduce the activity of α-glucosidase. Therefore, the inhibition of compounds **10d**, **12d**, **15d**, and embelin against α-glucosidase is reversible.

**Figure 6.**
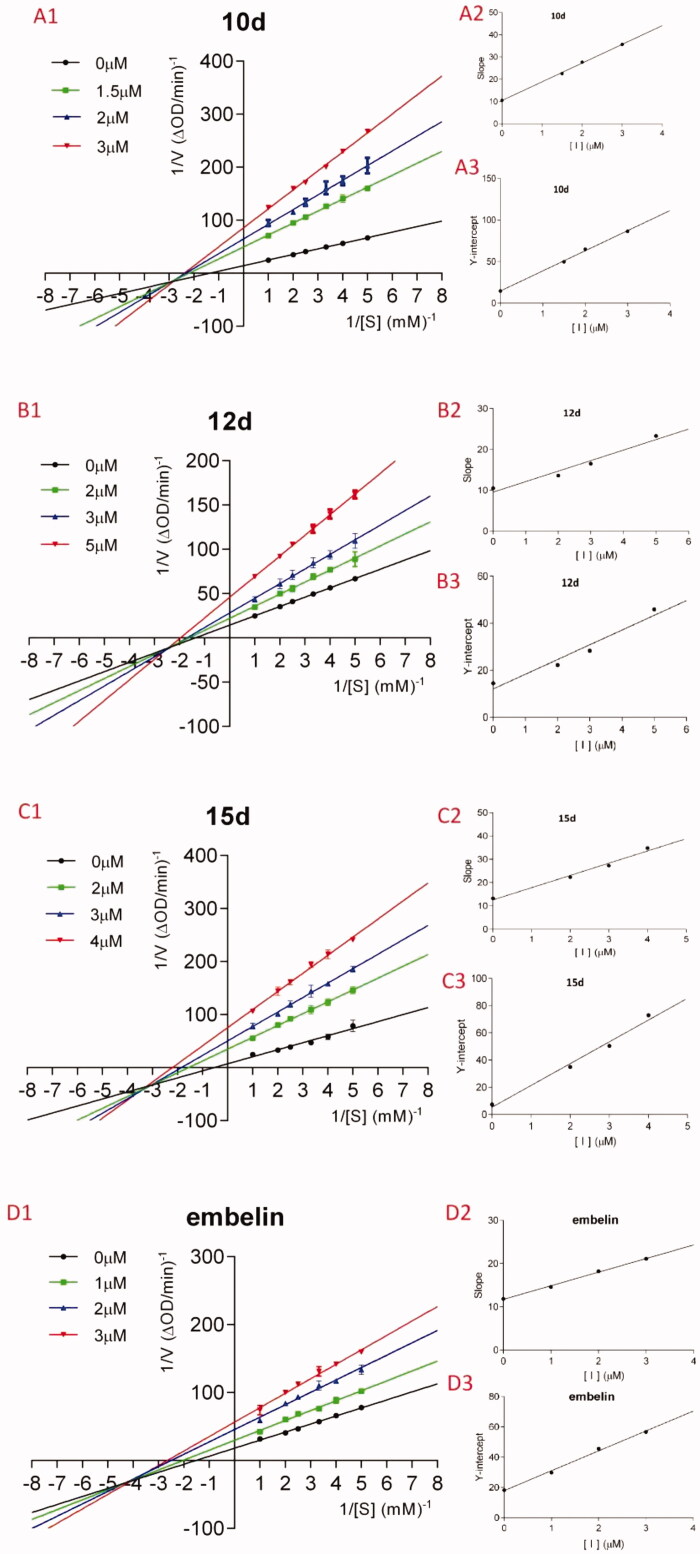
A1–D1: Lineweaver–Burk double-reciprocal plots; A2–D2: Plots of slope *versus* concentration of inhibitors for the determination of the inhibition constant K_i_; A3–D3: Plots of Y-intercept *versus* concentration of inhibitors for the determination of the inhibition constant K_is_.

In order to elucidate the action mode, the most potent compounds **10d**, **12d**, **15d**, and embelin were selected for enzyme kinetic studies. The kinetic data were expressed by Lineweaver–Burk double-reciprocal plots. As shown in Figure 6, the plots of 1/*v versus* 1/[*S*] give a group of straight lines with different slopes that intersect at the third quadrant (Figure 6(A1–D1)), suggesting that all of them are mixed-type inhibitors. Thus, these compounds bind not only with the free α-glucosidase but also with the α-glucosidase-PNPG complex. In other words, they inhibit the function of α-glucosidase by not only directly binding to free enzyme (EI), but also by interfering with the formation of the α-glucosidase-PNPG (ES) intermediate through producing an α-glucosidase-PNPG-inhibitor (ESI) complex in a non-competitive manner^33^. The inhibition constant for the inhibitor binding with free enzyme (K_i_) was determined from a plot of the slope (K_m_/V_m_) *versus* the inhibitor concentration (Figure 6(A2–D2)), and the inhibition constant for the inhibitor binding with enzyme–substrate complex (K_is_) was obtained from the vertical intercept (1/V_m_) *versus* the inhibitor concentration (Figure 6(A3–D3))^34^. The results are collected in Table 3: the K_is_ values of all of them are smaller than their K_i_ values, which mean that they have higher affinity with the enzyme-substrate complex than with the free enzyme.

**Table 3.** K_i_, K_is_, and inhibition type of selected compounds against α-glucosidase.

Compound	K_i_ value (μM)	K_is_ value (μM)	Inhibition type
Embelin	3.72	1.37	Mixed-type
**10d**	1.24	0.60	Mixed-type
**12d**	3.71	1.93	Mixed-type
**15d**	2.40	0.33	Mixed-type

### Molecular docking

**Figure 7.**
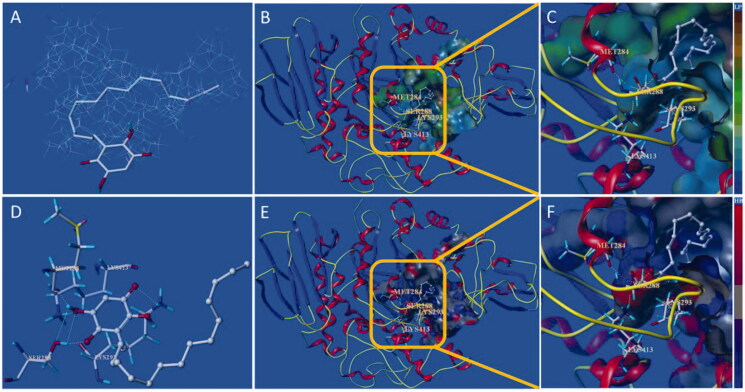
Docking binding model of **10d** with yeast α-glucosidase. (A): Binding mode of **10d** docked with the prototype molecular of the active site. (B) and (C): Active site MOLCAD surface representation of lipophilic potential. (D): The interaction of **10d** with the surrounding amino acids. (E) and (F): Active site MOLCAD surface representation of hydrogen bonding.

Compound **10d** and **15d** have the strongest inhibition against free α-glucosidase (Table 3), and molecular docking was introduced to confirm the binding mode of the two compounds with the active site of α-glucosidase. The protomol of the active site integrate with **10d** is as shown in Figure 7(A), which reveals that compound **10d** can be effectively inserted into the protomol. The results show that the long-chain *n*-C_15_H_31_ group can be inserted into the hydrophobic region of the active site selectively (Figure 7(B,C)). The hydrophobic pocket is long and relatively narrow, which explain why long-chain substituents in the 3-position are highly preferred. As depicted in Figure 7(D–F), the two carbonyl groups and two hydroxyl groups from *para*-benzoquinone core structure interact with several amino acid residues including MET284, LYS413, SER288, and LYS293, which locate in the entrance of the active pocket. These hydrophilic interactions reduce the binding free energy of inhibitor and target protein, resulting in the increase of inhibitory function. Thus, the hydroxyl groups of embelin and its derivatives are more helpful to improve the inhibitory activity against α-glucosidase, which also is in accordance with the *in vitro* assay.

**Figure 8.**
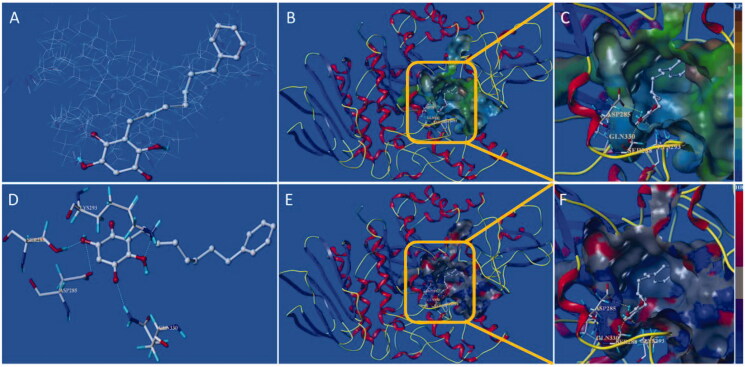
Docking binding model of **15d** with yeast α-glucosidase. (A): Binding mode of **15d** docked with the prototype molecular of the active site. (B) and (C): Active site MOLCAD surface representation of lipophilic potential. (D): The interaction of **15d** with the surrounding amino acids. (E) and (F): Active site MOLCAD surface representation of hydrogen bonding.

The docking binding mode of **15d** with α-glucosidase is shown in Figure 8. The results suggest that **10d** and **15d** have similar binding mode with the active site. However, the flexibility of the substituent (*n*-heptylphenyl) in the 3-position of **15d** is less than that of **10d** (*n*-pentadecyl), thus the hydrophobic portion of **15d** only interact with a part of the active hydrophobic pocket, while **10d** is more effective of interaction with the active site. This difference may be contributed to **10d** possessing more potent inhibitory activity.

## Conclusions

Embelin was first discovered to have potent inhibitory activity against α-glucosidase in this study. Based on the structure of embelin, four series of embelin derivatives were designed and prepared. All final compounds were characterised by ^1^H-NMR, ^13^C-NMR, and HRMS. Enzyme inhibition bioassay results show that most of these embelin derivatives have potent inhibitory activity. The SAR study suggests that the two hydroxyl groups and long-chain substituents are the key factors affected the inhibitory activity. The most active derivatives **10d**, **12d**, **15d**, and embelin were selected for the further mechanism and kinetic study. The results reveal all of them are reversible and mixed-type inhibitors. Molecular docking study was introduced to insight into the binding mode of **10d** and **15d** with α-glucosidase. The docking results suggest that the formation of hydrogen bonds between the hydrophilic groups and several amino acids at the active site, as well as the hydrophobic interaction between the hydrophobic substituents in the 3-position of *para*-benzoquinone and hydrophobic pocket, are important mechanism of inhibition. This study provides several leading structures for designing and developing novel AGIs. These findings encourage us to continue our efforts towards the optimisation of the pharmacological profile of these embelin derivatives.
